# A Kalman Filter-Based Localization Calibration Method Optimized by Reinforcement Learning and Information Matrix Fusion

**DOI:** 10.3390/e27080821

**Published:** 2025-08-01

**Authors:** Zijia Huang, Qiushi Xu, Menghao Sun, Xuzhen Zhu

**Affiliations:** 1 National Key Laboratory of Multi-Domain Data Collaborative Processing and Control, Xi’an 710068, China; beiyouhzj@163.com; 2 State Key Laboratory of Networking and Switching Technology, Beijing University of Posts and Telecommunications, Beijing 100876, China; 1518668454@bupt.edu.cn (Q.X.); 2024110214@bupt.cn (M.S.)

**Keywords:** Kalman filtering, reinforcement learning, collaborative correction

## Abstract

To address the degradation in localization accuracy caused by insufficient robustness of filter parameters and inefficient multi-trajectory data fusion in dynamic environments, this paper proposes a Kalman filter-based localization calibration method optimized by reinforcement learning and information matrix fusion (RL-IMKF). An actor–critic reinforcement learning network is designed to adaptively adjust the state covariance matrix, enhancing the Kalman filter’s adaptability to environmental changes. Meanwhile, a multi-trajectory information matrix fusion strategy is introduced, which aggregates multiple trajectories in the information domain via weighted inverse covariance matrices to suppress error propagation and improve system consistency. Experiments using both simulated and real-world sensor data demonstrate that the proposed method outperforms traditional extended Kalman filter approaches in terms of localization accuracy and stability, providing a novel solution for cooperative localization calibration of unmanned aerial vehicle (UAV) swarms in dynamic environments.

## 1. Introduction

With the rapid development of intelligent unmanned systems, the application of high-precision positioning technology for unmanned clusters has become a significant challenge. In dynamic scenarios, single sensors like Global Positioning System (GPS) often suffer from signal blockage and multipath effects, leading to degraded accuracy [1,2], while inertial measurement units (IMUs) accumulate errors over time and cannot support long-term localization independently [3]. Therefore, Kalman filter (KF)-based [4] multi-sensor fusion has become a mainstream solution, offering optimal estimation through a state-space model and theoretically enabling error minimization and calibration. However, the traditional Kalman filter relies on fixed parameters [5], leading to model mismatch in scenarios with motion state transitions (e.g., sudden acceleration, complex terrains) or time-varying sensor noise, which degrades localization accuracy—particularly in multi-trajectory cooperative scenarios.

In recent years, the localization calibration field has witnessed significant developments in both filtering models and learning-based strategies. Traditional Kalman filter variants, such as the extended Kalman filter (EKF) [6], unscented Kalman filter (UKF) [7], and particle filter (PF) [8], have been widely applied in multi-sensor fusion scenarios. However, these methods still face challenges in coping with non-Gaussian noise and dynamic model mismatch, particularly in rapidly changing environments. On the other hand, adaptive filtering approaches like the adaptive Kalman filter (AKF) [9] and adaptive noise-adjusted Kalman filter (ANKF) [10] attempt to adjust filter parameters online but often rely on fixed rule-based heuristics that limit their generalizability. More recently, reinforcement learning (RL) [11] and deep neural networks have been introduced to enhance filter adaptability and learn optimal correction policies from data. Representative examples include the reinforcement learning-based adaptive Kalman filtering algorithm (RL-AKF) [12] methods, which estimate noise covariances using policy networks. Nevertheless, most of these methods focus solely on single-trajectory filtering, lacking a cooperative perspective. Meanwhile, existing multi-platform fusion approaches tend to apply heuristic weighting without fully leveraging the structural properties of uncertainty [13].

Therefore, this study proposes a Kalman filter localization calibration method based on reinforcement learning optimization and information matrix fusion. An actor–critic network architecture [14] is constructed, where a gated recurrent unit (GRU) [15] captures temporal dependencies of sensor data, and an attention mechanism [16] focuses on key state features to adaptively generate KF parameter adjustment factors. These factors dynamically regulate the state covariance matrix. Since the state covariance matrix determines the relative confidence between prediction and observation, inaccurate covariance estimation may cause the filter to under- or over-trust measurements, leading to degraded accuracy or even divergence. Therefore, adaptive optimization of the covariance matrix is essential for improving filter robustness in dynamic and uncertain environments. Additionally, we introduce a multi-trajectory information matrix fusion algorithm that aggregates inverse covariance matrices to form a layered uncertainty calibration mechanism. The main contributions of this paper are as follows: (1) Adaptive model uncertainty adjustment via reinforcement learning; (2) covariance-aware fusion for multi-trajectory cooperative localization; (3) significant accuracy improvements in both simulated and real-world UAV swarm data.

This paper is organized as follows: Section 2 reviews existing calibration methods; Section 3 details the algorithm, including the network architecture, dynamic adjustment mechanism for state covariance, and the derivation of the fusion algorithm based on information matrix inversion. Section 4 presents the experimental design and results. Section 5 concludes the study and discusses future work and applications.

## 2. Related Work

At present, multi-sensor fusion has become the mainstream approach for localization calibration in UAV swarms. Among them, GPS/IMU-integrated navigation systems are widely used due to their complementary advantages [17,18]. The KF, as a classical algorithm, performs optimal recursive estimation for linear systems. To address the significant errors introduced when using the KF in nonlinear scenarios [19], several nonlinear filtering variants have been developed, including the EKF, UKF, and PF. Moreover, to improve filter stability in high-dynamic environments, adaptive variants such as the AKF and federated Kalman filter (FKF) [20] have been proposed. These evolutionary filtering methods provide theoretical support for achieving high-precision localization in UAV swarms operating under complex environments, aggressive maneuvers, and sensor failures. In addition to classical adaptive Kalman filtering techniques, several advanced approaches have been proposed to address model uncertainty. These include interacting multiple-model (IMM) filters that handle mode switching [21], set-membership-based hybrid filters that enforce bounded state uncertainty [22], and robust cubature Kalman filters for tightly coupled GNSS/INS systems [23]. Compared to these methods, our approach employs reinforcement learning to adaptively scale the state covariance based on temporal observation patterns, without relying on predefined model structures.

In the development of integrated navigation technologies, machine learning-based adaptive calibration methods have gradually emerged in recent years with the advancement of deep learning and reinforcement learning [24,25]. These methods leverage historical data to learn complex error patterns and achieve high-precision calibration. For example, Wei et al. [26] proposed a navigation method that integrates random forest regression with adaptive Kalman filtering to improve accuracy under limited data availability. Liu et al. [27] proposed an integrated navigation filtering algorithm assisted by a radial basis function (RBF) neural network, aiming to improve the navigation accuracy of the system in the case of GPS signal loss. Other studies [28] have combined deep learning with extended state Kalman filtering (ES-EKF), using Long Short-Term Memory (LSTM) [29] networks to capture temporal dependencies in sensor data and convolutional neural networks (CNNs) [30] to extract spatial features. Furthermore, the RL-AKF has been proposed, which adaptively estimates the process noise covariance using reinforcement learning techniques. In the Kalman filter framework, the state covariance matrix plays a central role in quantifying uncertainty and directly affects the computation of the Kalman gain. However, current methods still largely rely on traditional techniques for its adaptive adjustment. Notably, sliding window techniques have been widely used to enhance temporal adaptability in dynamic filtering. For instance, the moving-window-based adaptive fitting H∞ filter [31] leverages windowed data to suppress nonlinear system disturbances by focusing on recent state transitions, while the Sage windowing and random weighting adaptive filtering method [32,33] mitigates kinematic model errors through discounted weighting of older data within a sliding window. These methods demonstrate the effectiveness of temporal local processing in dynamic uncertainty quantification. Therefore, this paper proposes a reinforcement learning-based approach using an actor–critic architecture to dynamically adjust the state covariance matrix.

Meanwhile, in the area of multi-trajectory cooperative calibration, inter-platform information exchange is a critical component for achieving high-precision collaboration. Existing mainstream methods focus on fusion strategies for heterogeneous multi-source data. For example, in cooperative localization of UAV swarms, platforms can share position and inertial data; ultra-wideband (UWB) ranging technology is used to acquire relative distance constraints [34]; angle-of-arrival (AOA) methods employ antenna arrays to determine signal directions [35], forming joint constraints in distance and angle; and vision sensors can be used to capture environmental features or visual markers of other UAVs, with visual SLAM algorithms employed to extract feature correspondences and construct relative pose estimates [36]. Furthermore, cutting-edge fusion techniques such as random weighting estimation have been applied to multi-dimensional data fusion, dynamically adjusting weights based on real-time data credibility to enhance robustness under varying noise conditions [37]. The random weighting method for multi-sensor data fusion, known for its flexibility in handling unknown noise distributions, has also been adopted to improve fusion accuracy in heterogeneous sensor networks [38]. For complex integrated systems like INS/GNSS/CNS, matrix-weighted multi-sensor fusion strategies optimize performance by leveraging adaptive weight matrices that account for cross-correlations between sensors, ensuring more reliable state estimation [39]. Notably, several works (e.g., Li et al. [40]) have addressed uncertainty quantification in multi-sensor fusion. However, relatively fewer studies address real-time, decentralized fusion of state covariance across multiple moving platforms under Kalman filtering frameworks. Therefore, this study proposes a cooperative calibration algorithm based on multi-trajectory information matrix fusion. This approach enables a more precise characterization of uncertainty distribution across trajectories in dynamic environments and provides a novel solution for high-precision cooperative localization of UAV swarms in complex scenarios.

## 3. Methods

**Figure 1:**
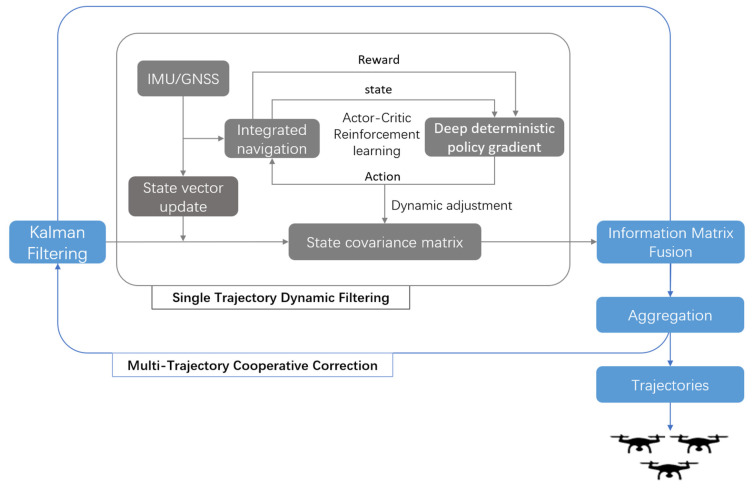
The framework adopts a “single-trajectory dynamic filtering–multi-trajectory collaborative correction” architecture. On the left is the single-trajectory Kalman filter parameter optimization process driven by the actor–critic reinforcement learning network, while on the right is the collaborative correction mechanism based on multi-trajectory information matrix fusion. By dynamically adjusting the state covariance matrix and aggregating information matrices, the framework achieves hierarchical calibration of positioning errors.

To address the challenges of trajectory parameter uncertainty quantification and dynamic calibration in cooperative localization of UAV swarms, this study proposes a collaborative localization framework that integrates reinforcement learning with information matrix fusion. A dual closed-loop architecture—“single-trajectory dynamic filtering and multi-trajectory cooperative correction”—is adopted (as shown in Figure 1). First, an actor–critic reinforcement learning network is used to adaptively optimize the state covariance matrix in the Kalman filter for each individual trajectory. Then, an information matrix fusion strategy is applied, in which each trajectory’s covariance matrix is transformed into its corresponding information matrix. By aggregating these matrices, the framework fuses the credibility of multi-source trajectory estimates and suppresses error propagation from individual platforms. The overall algorithm consists of two key components: reinforcement learning-driven filter parameter optimization and multi-trajectory information matrix fusion.

### 3.1. Reinforcement Learning-Driven Filter Parameter Optimization

In the state update process of the Kalman filter, this study employs a reinforcement learning mechanism to dynamically adjust the state covariance matrix.

We first define the system state vector $x_{k}={\left[\begin{array}{cccc} p_{k} & v_{k} & q_{k} & a_{k} \end{array}\right]}^{T}$, including the platform’s position $p_{k}$, velocity $v_{k}$, quaternion $q_{k}$ (which belongs to the unit quaternion group isomorphic to SO(3)) [41], and acceleration $a_{k}$. Here, $q_{k}={\left[q_{w},\;q_{x},\;q_{y},\;q_{z}\right]}^{T}$ denotes the unit quaternion representing the platform’s orientation at time step *k*. It can be derived from the Euler angles (ϕ,θ,ψ), corresponding to roll, pitch, and yaw, respectively, as

(1) $$q_{k}=\left[\begin{array}{c} q_{w}\\ q_{x}\\ q_{y}\\ q_{z} \end{array}\right]=\left[\begin{array}{c} \cos(\phi /2)\cos(\theta /2)\cos(\psi /2)+\sin(\phi /2)\sin(\theta /2)\sin(\psi /2)\\ \sin(\phi /2)\cos(\theta /2)\cos(\psi /2)-\cos(\phi /2)\sin(\theta /2)\sin(\psi /2)\\ \cos(\phi /2)\sin(\theta /2)\cos(\psi /2)+\sin(\phi /2)\cos(\theta /2)\sin(\psi /2)\\ \cos(\phi /2)\cos(\theta /2)\sin(\psi /2)-\sin(\phi /2)\sin(\theta /2)\cos(\psi /2) \end{array}\right]$$

Note that gyroscope measurements are not included in the state vector, as they are treated as control inputs that drive the quaternion update through the angular velocity term in the state transition function. The state update is as follows:

(2) $$x_{k+1}=f\left(x_{k},u_{k}\right)+w_{k}$$

(3) $$P_{k+1}=F_{k}P_{k}{F_{k}}^{T}+Q_{k}$$

Here, $x_{k+1}$ and $P_{k+1}$ are the system state vector and covariance matrix at time step k+1; $w_{k}$ is the process noise, which is assumed to be zero-mean Gaussian white noise with covariance matrices $Q_{k}$; and $F_{k}$ is the Jacobian of the nonlinear state transition function f(·). In our approach, the process noise covariance $Q_{k}$ is initially set as a prior based on sensor characteristics, $Q_{k}=\mathrm{diag}\left(Q_{pp},Q_{vv},Q_{qq},Q_{aa}\right)$, where $Q_{pp}=\sigma_{p}^{2}I_{3}$, $Q_{vv}=\frac{dt^{2}}{2}Q_{aa}$, $Q_{qq}={\left(\frac{ARW\cdot \pi }{180\cdot \sqrt{3600}}\cdot dt\right)}^{2}I_{4}$(ARW: angular random walk), $Q_{aa}={\left(\frac{VRW\cdot 9.81}{\sqrt{3600}}\cdot dt\right)}^{2}I_{3}$(VRW: velocity random walk) (the specific sensor parameters are introduced in Section 4.1), but it may not fully reflect real-time dynamics or noise variations. While $Q_{k}$ affects Equation (2), state covariance P better captures real-time uncertainties that Q cannot. Optimizing P via reinforcement learning adapts to these time-varying factors, complementing a fixed Q for more accurate uncertainty quantification. The function $f(x_{k},u_{k})$ defines the motion dynamics of the UAV platform and is given as

(4) $$f\left(x_{k},u_{k}\right)=\left[\begin{array}{c} p_{k}+v_{k}\cdot dt+0.5\cdot a_{k}\cdot dt^{2}\\ v_{k}+a_{k}\cdot dt\\ \mathrm{QuaternionUpdate}(q_{k},\omega_{k},dt)\\ a_{k} \end{array}\right]$$

where $\omega_{k}$ is the angular velocity measured by the gyroscope, and the quaternion is updated based on standard quaternion kinematics driven by $\omega_{k}$. The quaternion update follows the exponential map on $SO\left(3\right):q_{k+1}=q_{k}⊗\exp\left(\frac{dt}{2}\omega_{k}\right)$, where $\left(\exp:R^{3}\to S^{3}\right)$ is the quaternion exponential map, and ⊗ denotes quaternion multiplication [42].

The Jacobian matrix $F_{k}$ of the state transition function f(·) includes the dynamic coupling terms among position, velocity, and acceleration, as well as quaternion updates, where $\Omega \left(\omega \right)=\left[\begin{array}{cccc} 0 & -\omega_{x} & -\omega_{y} & -\omega_{z}\\ \omega_{x} & 0 & \omega_{z} & -\omega_{y}\\ \omega_{y} & -\omega_{z} & 0 & \omega_{x}\\ \omega_{z} & \omega_{y} & -\omega_{x} & 0 \end{array}\right]$ is an antisymmetric matrix constructed from angular velocity:

(5) $$F_{k}=\left[\begin{array}{cccc} I_{3} & dt\cdot I_{3} & 0_{3\times 4} & 0.5\cdot dt^{2}\cdot I_{3}\\ 0_{3\times 3} & I_{3} & 0_{3\times 4} & dt\cdot I_{3}\\ 0_{4\times 3} & 0_{4\times 3} & F_{q} & 0_{4\times 3}\\ 0_{3\times 3} & 0_{3\times 3} & 0_{3\times 4} & I_{3} \end{array}\right]\;$$

(6) $$F_{q}=\left\{\begin{array}{c} I_{4}+\frac{dt}{2}\cdot \Omega \left(\omega \right),||\omega ||\ll 1\;\\ \cos\left(\frac{||\omega ||\cdot dt}{2}\right)I_{4}+\frac{\sin(\frac{||\omega ||\cdot dt}{2})}{\left||\omega |\right|}\Omega \left(\omega \right),others \end{array}\right.$$

Specifically, the block structure of F is constructed by linearizing the nonlinear state transition function around the current state, considering the temporal derivatives (with time step dt) of position with respect to velocity and acceleration, and the quaternion update law using the antisymmetric matrix Ω(ω) of angular velocity ω. The quaternion update block $F_{q}$ in Equation (4) is derived from the kinematic equation of quaternions under rotational motion: for small angular velocities (∥ω∥≪1), it approximates the first-order Taylor expansion of the quaternion exponential map; for general cases, it uses the exact form of quaternion rotation based on trigonometric functions, consistent with standard quaternion update rules in inertial navigation [43].

The observation model is given by

(7) $$z_{k}=Hx_{k}+v_{k}$$

where $z_{k}$ is the GPS measurement vector (3D position), explicitly given by $z_{k}={\left[x_{\mathrm{GPS}},y_{\mathrm{GPS}},z_{\mathrm{GPS}}\right]}^{T}$; $H=\left[\begin{array}{cccc} I_{3\times 3} & O_{3\times 3} & O_{3\times 4} & O_{3\times 3} \end{array}\right]$ is the observation matrix; and $v_{k}$ is measurement noise, which is assumed to be zero-mean Gaussian white noise with covariance matrices *R*. *H* is defined as the observation Jacobian, a linear mapping that selects the position components from the full state vector. Observations are position measurements from GPS, while IMU data (accelerometer, gyroscope) serve as inputs to the dynamic motion model in Equation (1) to drive state transitions. The innovation vector is then defined as $y_{k}=z_{k}-Hx_{k}$.

Then, we calculate the Kalman gain $K_{k}$, which represents the degree of influence of the observed values on the state update:

(8) $$K_{k}=P_{k}H_{k}^{T}{\left(HP_{k}H^{T}+R\right)}^{-1}$$

where *R* represents the measurement noise covariance matrix, quantifying the uncertainty of GPS position measurements. It is initialized as a diagonal matrix based on the known noise characteristics of the GPS sensor. Specifically, we set $R=\mathrm{diag}(\left[\sigma_{x}^{2},\sigma_{y}^{2},\sigma_{z}^{2}\right])$, where $\sigma_{x}=\sigma_{y}=\sigma_{z}\approx 2.5\;m$ (converted from CEP50 to standard deviation for Gaussian noise modeling).

Finally, we update the state vector based on innovation $y_{k}$ and Kalman gain:

(9) $${\hat{x}}_{k}=x_{k}⊕K_{k}y_{k}$$

To enable the Kalman filter to adaptively adjust its covariance in response to dynamic environmental changes, we introduce an RL module between the prediction and update steps. Specifically, the RL agent observes a sliding window of past states, extracts temporal features through a GRU-attention mechanism, and outputs a scaling factor that adjusts the state covariance matrix before computing the Kalman gain. This adjustment loop forms a closed process where the RL policy is iteratively optimized based on the positioning error after each update, enabling real-time adaptation of the filter to varying motion conditions.

For reinforcement learning modeling, the actor network employs a two-layer GRU to extract historical state sequences within a sliding window $S_{k}=\left[\begin{array}{ccc} x_{k-n}, & \ldots , & x_{k} \end{array}\right]$(*n* = 10). The sliding window is used to capture temporal local dependencies in dynamic UAV motion: recent states (within the window) retain critical information about short-term motion continuity (e.g., acceleration trends, attitude transitions) while excluding overly distant data that may introduce noise from outdated motion patterns. An attention mechanism is applied to assign weights to key frame features:

(10) $$\alpha_{k}=softmax\left(W_{2}\tanh\left(W_{1}\cdot GRU\left(S_{k}\right)\right)\right),c_{k}=\sum \alpha_{k}\cdot GRU\left(S_{k}\right).$$

In both the actor and critic networks, the attention mechanism assigns weights to key frame features of the state sequence extracted by the GRU. This enhances capture of important temporal dependencies in sensor data, improving the actor’s dynamic adjustment of covariance matrices and the critic’s precision in state-value estimation.

The policy network generates covariance adjustment parameters based on the contextual feature $c_{k}$. It outputs the mean μ and standard deviation σ through fully connected layers to define a Gaussian distribution $a_{k}Ñ(\mu ,\sigma^{2})$, from which an action $a_{k}$ is sampled. This action is then used to update the state covariance matrix: $P_{k|k}=P_{k|k}\cdot abs\left(a_{k}\right)$. Here, abs(.) denotes the element-wise absolute value to ensure positive scaling of the covariance matrix. The critic also employs a GRU combined with an attention mechanism to extract temporal features and evaluate the state value V(s), and uses the negative Euclidean distance between the estimated and true positions $r=-\left|\right|{\hat{x}}_{k}-x_{k}^{true}\left|\right|$ as the reward. Where *V(s)* represents the estimated value of the current system state and this value estimation reflects the long-term cumulative reward expected from the current state under the optimal policy, guiding the actor network to adjust the state covariance matrix more effectively.

The policy update process optimizes the network parameters using the advantage function, where γ=0.99 is the discount factor:

(11) $$A_{k}=r_{k}+\gamma V\left(s_{k+1}\right)-V\left(s_{k}\right)$$

The parameters of the actor network are updated using the following gradient:

(12) $$\nabla_{\theta }J\left(\theta \right)=E_{k}\left[\nabla_{\theta }\log\pi \left(a_{k}|s_{k};\theta \right)\cdot A_{k}\right]$$

The parameters of the critic network are updated by minimizing the mean squared error (MSE):

(13) $$L_{critic}={\left(r_{k}+\gamma V\left(s_{k+1}\right)-V\left(s_{k}\right)\right)}^{2}$$

**Figure 2:**
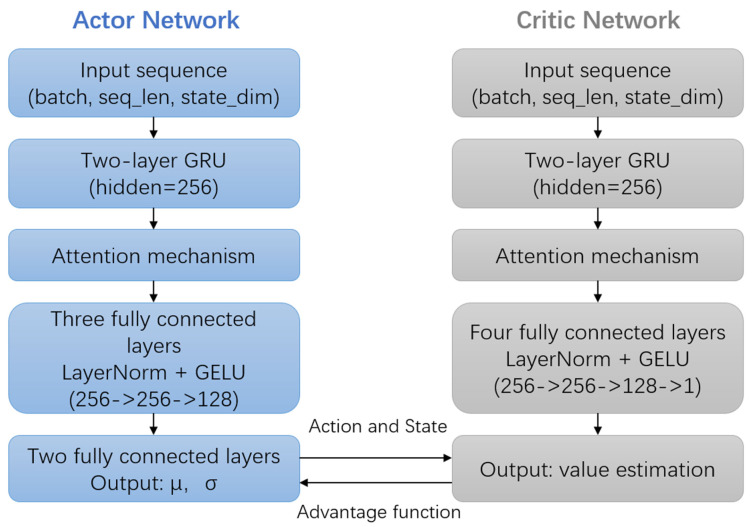
Both the actor and critic networks take the state sequence within a sliding window as input. Temporal features are extracted through two layers of a GRU, and an attention mechanism is applied to focus on key states. The actor network outputs covariance adjustment parameters (mean μ and standard deviation σ), while the critic network outputs the estimated state value. Policy optimization is achieved through the advantage function.

The overall network architecture is illustrated in Figure 2 below. Unlike simple scaling, this framework adjusts each dimension (adjustments to 13 dimensions) of P based on real-time motion dynamics. As detailed in Equations (11)–(13) and Figure 2, the agent learns dimension-specific adjustment strategies by analyzing motion characteristics, ensuring each sub-block of P accurately reflects the actual noise level of its corresponding state component, thereby enhancing the reliability of state estimation.

### 3.2. Multi-Trajectory Information Fusion

At the multi-platform level, to achieve cooperative correction of trajectory information among platforms, this paper proposes a fusion strategy based on the information matrix.

First, for trajectory *i*, its state covariance matrix is $P_{i}$ and the corresponding information matrix is defined as $\Lambda_{i}=P_{i}^{-1}$ (in practice, a pseudo-inverse is used to replace the inverse for singular matrices). For the entire UAV swarm, the fused information matrix is given by

(14) $$\Lambda_{fused}=\sum \Lambda_{i}$$

(15) $$P_{fused}=\Lambda_{fused}^{-1}$$

Considering the differences in covariance among trajectories, the following method is used to compute the mean in order to avoid distortion:

(16) $$P_{mean}=\frac{1}{N}\sum_{i=1}^{n}P_{i}$$

Using the norm $\left|\right|P_{mean}\left|\right|$ as a reference threshold, an asymmetric correction mechanism is adopted. For each trajectory i, if its covariance norm $\left|\right|P_{i}\left||>|\right|P_{mean}\left|\right|$, the trajectory is considered to have high uncertainty. In this case, the fused result is fed back to the original trajectory and updated by weighted averaging:

(17) $$P_{i}=\frac{1}{2}\left(P_{mean}+P_{i}\right),||P_{i}\left||>|\right|P_{mean}\left|\right|$$

This enables trajectory estimates with high uncertainty to have their covariance compressed using the fused result, thereby improving overall estimation consistency and system robustness while avoiding the degradation of high-quality estimates.

## 4. Experiments and Results

### 4.1. Experimental Setup

**Figure 3:**
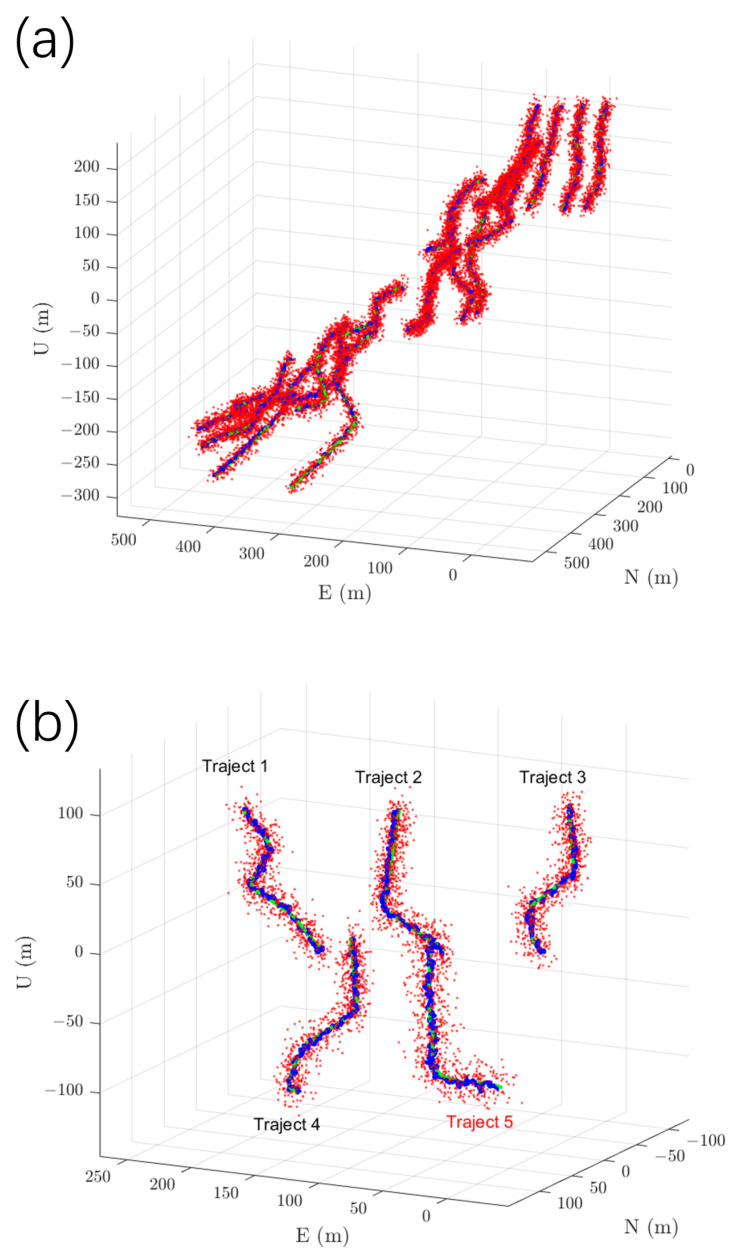
Visualization of training and testing trajectories. (**a**) shows 20 sets of training trajectories, and (**b**) shows 5 sets of testing trajectories (trajectory 5 represents real-world data collected by the WTGAHRS3-232 module). Red dots indicate GPS observations, the green curve represents the ground truth without error, and the blue curve shows the RL-IMKF estimated trajectories, illustrating motion under various dynamic scenarios.

The experiments use the IMU data and GNSS signals generated by the simulation software GNSS-INS-SIM (Version 2.1), which produces IMU data and GPS signals with configurable noise characteristics (consistent with typical low-cost sensors: gyroscope (bias drift 10°/h, ARW 0.75°/$\sqrt{h})$ and accelerometer (bias drift 2 × 10^−4^ g, VRW 0.05 g/$\sqrt{h}$). The dataset is divided into training and testing sets, covering motion scenarios including static, uniform motion, and accelerated turns. The training dataset contains 20 trajectories covering multiple dynamic motion patterns; the testing dataset contains 5 independent trajectories not included in training, used to evaluate the algorithm’s generalization capability. Additionally, a set of real data collected by the WTGAHRS3-232 (sourced from WitMotion Shenzhen Co., Ltd., Shenzhen, China) inertial measurement module is introduced for validation, whose main parameters are accelerometer range: ±16 g; RMS noise: 0.75 1mg-rms; gyroscope range: ±2000°/s; RMS noise: 0.05°/s-rms; attitude accuracy (pitch/roll): 0.2°; GPS positioning accuracy: 2.5 m CEP50. During algorithm training, the actor–critic network uses a batch size of 20, learning rates of 1 × 10^−5^ for the actor and 1 × 10^−4^ for the critic, and a temporal window length where sequence length = 10. The Adam optimizer is used for 20 training epochs. The evaluation metric is the three-dimensional root mean square error (RMSE) of position. The effectiveness of the proposed algorithm is validated by comparing its results with those of the EKF and other methods. Figure 3 below shows the 20 training trajectories and 5 testing trajectories. Trajectories 1–4 are simulated data, while trajectory 5 is the ground truth data. Red scatter points represent GPS observations, the green curve denotes the error-free ground truth, and the blue curve indicates the estimated trajectory.

### 4.2. Experimental Methods

**Figure 4:**
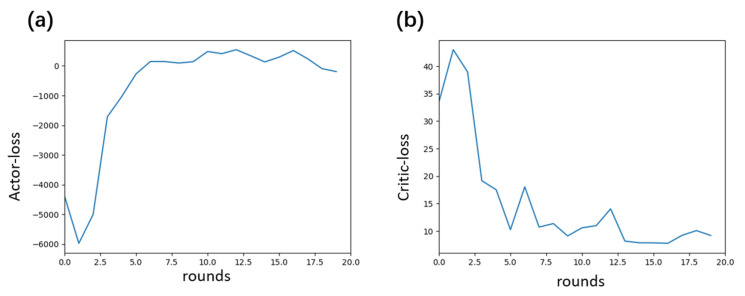
Loss curves during the training process. (**a**) The actor loss exhibits a three-phase convergence pattern: significant fluctuations during the initial random exploration phase, a rapid increase during the policy optimization phase, and eventual stabilization near zero. (**b**) The critic loss decreases from a high level to low-amplitude fluctuations as the value function fitting accuracy improves, reflecting the progressively improving accuracy of state-value estimation.

The experiment was conducted through the following steps for model training and testing analysis. During the training phase, simulated data was used to construct state sequences (13 dimensions including position, velocity, quaternion, and acceleration). The actor–critic network optimized the dynamic adjustment parameters of the Kalman filter covariance matrix, using the negative position error as the reward function. Training proceeded iteratively until the loss function converged. During testing, the trained model was applied to both simulated and real data to calculate the error between the filtered trajectory positions and the ground truth, and to compare the performance of the proposed method against other filtering methods. Figure 4a below shows the actor loss trend during training, which exhibits three distinct stages: initially, due to random parameters and exploratory strategies, the loss fluctuated sharply; subsequently, as the network learned effective strategies, the loss rapidly increased above zero and the parameter adjustment direction became clearer; finally, the loss stabilized around zero with minor fluctuations, indicating ongoing fine-tuning. In Figure 4b, the critic loss decreased rapidly at first due to large value estimation errors, then fluctuated under data and scenario disturbances, and eventually converged to a low level with small oscillations, reflecting progressively more accurate value fitting.

### 4.3. Evaluation of Experimental Results

**Figure 5:**
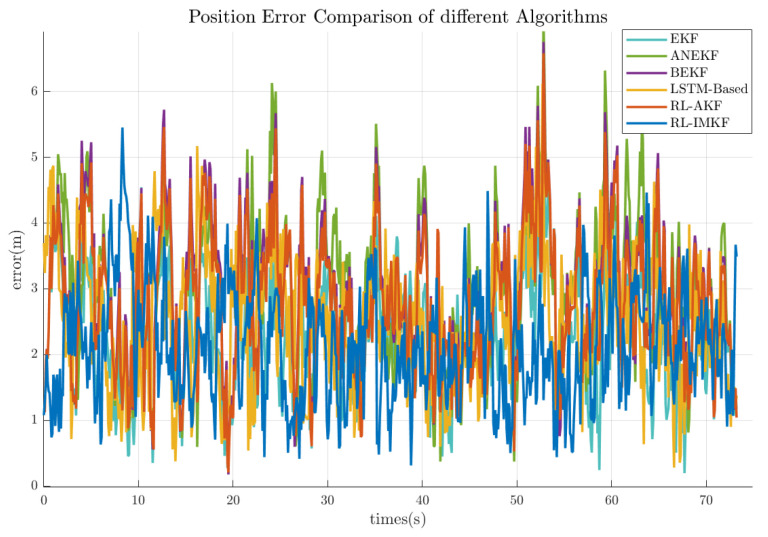
Temporal comparison of positioning errors across different algorithms. This compares the positioning errors of EKF, ANKF, BEKF, LSTM-based method, RL-AKF, and RL-IMKF in dynamic scenarios. The RL-IMKF error curve (blue) exhibits the highest overall smoothness, with peak errors significantly lower than those of the other algorithms, demonstrating the stability advantages of dynamic covariance optimization and information matrix fusion.

To evaluate the performance of the different filtering methods, this experiment compared the standard EKF, adaptive noise-adjusted Kalman filter (ANKF) [44], bidirectional extended Kalman filter (BEKF) [45], LSTM-based trajectory estimation algorithm [46], adaptive Kalman filter algorithm based on reinforcement learning, and the proposed RL-IMKF, where a number of trajectories M = 5 was used for the information matrix fusion. The primary comparison metric was the average position error across each trajectory. Table 1 lists the average position errors for the different filtering methods. The RL-IMKF method demonstrated the lowest error, achieving a 17.4% reduction compared to EKF. This validates that RL-IMKF improves system stability by dynamically generating covariance scaling factors through the actor–critic network. Figure 5 illustrates the positional error trends for the same trajectory under different algorithms. The RL-IMKF shows a smoother overall error curve, with both peak values and fluctuation amplitudes smaller than the methods, demonstrating a clear advantage in stability.

The experimental results indicate that the standard EKF, due to its use of fixed filtering parameters, is prone to model mismatch in dynamic motion scenarios, resulting in the largest errors. Although ANKF introduces an adaptive noise adjustment mechanism, it relies on predefined noise variation models, limiting its adaptability to complex motion patterns. BEKF improves trajectory smoothness through bidirectional filtering but does not consider the credibility differences among multiple trajectories. The LSTM-based trajectory estimation method leverages recurrent neural networks to model temporal dependencies in sequential positioning data, but the deterministic nature of LSTM predictions lacks explicit uncertainty quantification. RL-AKF uses reinforcement learning to adjust process noise but does not integrate multi-trajectory fusion, restricting its ability to suppress individual trajectory errors. In contrast, RL-IMKF dynamically optimizes the state covariance matrix of the Kalman filter through reinforcement learning and combines information matrix fusion to leverage multi-trajectory redundancy. This approach not only enables real-time filter parameter optimization but also suppresses error propagation from abnormal trajectories via the cumulative information matrix mechanism, demonstrating superior performance in both training data fitting and test data generalization.

Additionally, to investigate the impact of the number of trajectories on positioning accuracy in multi-trajectory fusion, an experiment selecting trajectory 1 compared the single-trajectory position errors of IMKF with trajectory counts, M, ranging from 1 to 5. The results are shown in Table 2.

It can be seen that when only a small number of trajectories are fused, the improvement in positioning accuracy is not significant, and error fluctuations may occur due to information redundancy; however, as the number of trajectories increases, the complementary information among multiple trajectories gradually dominates, resulting in a significant decrease in positioning error with the increase in the number of trajectories.

## 5. Conclusions

This study proposes a Kalman filter localization calibration method based on RL-IMKF, effectively addressing the robustness issue of filter model parameters in dynamic positioning of unmanned clusters. The constructed actor–critic network captures temporal features using a GRU and attention mechanisms to dynamically generate covariance adjustment factors. Simultaneously, a multi-trajectory information matrix fusion algorithm is designed to aggregate the inverse of the trajectory covariance matrices, quantify uncertainty, suppress error propagation, and improve the utilization of multi-source data. The experimental results demonstrate that RL-IMKF achieves the lowest average position error on both training and testing datasets, significantly reducing errors by 17.4% compared to traditional methods such as EKF. Regarding error variation trends, the RL-IMKF error curve is smoother, with peak values and fluctuation amplitudes noticeably smaller than those of other comparative algorithms. Furthermore, a dedicated experiment on the impact of the number of fused trajectories on positioning accuracy shows that reasonably increasing the number of fused trajectories leverages complementary information to reduce errors, providing valuable insights for practical engineering applications.

In practical applications, the RL-IMKF method demonstrates significant potential for adaptation to diverse scenarios. Whether deployed in intelligent unmanned system clusters with stringent environmental perception requirements or in complex environments affected by signal interference and data noise, this method achieves high-precision localization calibration through dynamic optimization of filter parameters and fusion of multi-source trajectory information. From transportation and logistics to monitoring and reconnaissance, RL-IMKF can enhance the reliability and cooperation of unmanned device positioning, facilitating broader and more effective deployment of unmanned systems across multiple fields.

In summary, the RL-IMKF method offers a new approach for high-precision positioning of unmanned clusters. Its strong performance in both theoretical validation and experimental testing provides technical support for the widespread application of intelligent unmanned systems in military, civilian, and other fields. Although this study has achieved significant progress, there remains room for optimization and further development. Future work could explore integrating multi-source heterogeneous sensor data such as UWB, vision, and LiDAR into the algorithm framework, and improve the design of the reinforcement learning state-space and information matrix fusion strategies to enable multimodal data collaboration in more complex environments. Additionally, considering the dynamic changes in communication topology during unmanned cluster movement, researching algorithms based on graph neural networks for trajectory association and dynamic fusion will also be a crucial direction to enhance algorithm adaptability.

## Figures and Tables

**Table 1:** Comparison of average positioning errors across different filtering methods.

Filtering Method	Average Error on Training Data (m)	Average Error on Test Data (m)
Raw GNSS	9.0915	9.8605
EKF	2.7469	2.9337
ANKF	2.6414	2.8140
BEKF	2.4125	2.7037
LSTM-based	2.4767	2.5836
RL-AKF	2.5756	2.6932
RL-IMKF	2.3141	2.4221

**Table 2:** Single-trajectory positioning errors under different numbers of fused trajectories M.

M	Position Error (m)
1	2.6679
2	2.6373
3	2.6655
4	2.1202
5	2.0767

## Data Availability

The data used in this study are available from the corresponding author upon reasonable request.
